# Complete chloroplast genome sequence of the *Pseudostellaria longipedicellata* S. Lee, K. Heo & S. C. Kim (Caryophyllaceae)

**DOI:** 10.1080/23802359.2018.1535840

**Published:** 2018-10-26

**Authors:** Yongsung Kim, Kyeong-In Heo, Sangtae Lee, Jongsun Park

**Affiliations:** a Infoboss Co., Ltd, Seoul, Korea;; b Infoboss Research Center, Seoul, Korea, 07766;; c Department of Biological Sciences, Sungkyunkwan University, Suwon, Korea

**Keywords:** word, *Pseudostellaria*, chloroplast genome, *Pseudostellaria longipedicellata*, Caryophyllaceae, Korean endemic species

## Abstract

*Pseudostellaria longipedicellata* which belongs to Caryophyllaceae is endemic in South Korea. In this study, we presented first complete chloroplast genome of *P. longipedicellata* which is 149,626 bp and has four sub-regions: 81,292 bp of large single copy (LSC) and 16,984 bp of small single copy (SSC) regions are separated by 25,675 bp of inverted repeat (IRs) regions including 126 genes (81 CDS, eight rRNAs, and 37 tRNAs). The overall GC contents of the chloroplast genome were 36.5% and in the LSC, SSC and IR regions were 34.3%, 29.3%, and 42.4%, respectively.

*Pseudostellaria* Pax (Caryophyllaceae) is a small genus distributed in temperate region. It consists of 25 species presenting high diversity in Asia. Two of three species distributed in North America have been separated into a new genus *Hartmaniella* M.L.Zhang & Rabeler but phylogenetic position of the other species *P. jamesiana* is still unclear forming a clade with *Stellaria americana*. Except these three species, core *Pseudostellaria* divided into two clades is monophyletic (Zhang et al. 2017).

*Pseudostellaria longipedicellata* S. Lee, K. Heo & S. C. Kim was first announced as new species in 2012(Lee et al. 2012). Morphological characters of *P. longipedicellata* are closely related to those of *Psedusotellaria palibiniana* and *Psedusotellaria okmotoi*. These are distinguished from *P. longipedicellata* by shorter pedicel and puberulent pedicels, respectively (Ohwi 1937; Choi 1999) and by being distributed allopatically between *P. longipedicellata* and the rest of species (unpublished data). Here, we presented the complete chloroplast genome sequence of *P. longipedicellata* as a first chloroplast genome in *Pseudostellaria* genus.

*Pseudostellaria longipedicellata* was collected in Mt. Taebaek, Gangwon Province, South Korea (Voucher in Cyber Herbarium at InfoBoss; Y. Kim, IB-00654). Total DNA was extracted from fresh leaves of *P. longipedicellata* using a DNeasy Plant Mini kit (QIAGEN, Hilden, Germany). Genome sequencing was performed using HiSeq 4000 (Macrogen Inc, Seoul, Korea), and *de novo* assembly was done using Velvet 1.2.21 (Zerbino and Birney 2008). Geneious R8 v8.0.5 (Biomatters Ltd, Auckland, New Zealand) was used for chloroplast genome annotation.

The chloroplast genome of *P. longipedicellata* (MH373593) is 149,626 bp and has four sub-regions: 81,292 bp of large single copy (LSC) and 16,984 bp of small single copy (SSC) regions are separated by 25,765 bp of inverted repeat (IRs). It contained 126 genes (81 CDS, 8 rRNAs, and 37 tRNAs); 18 genes (seven CDS, four rRNAs, and seven tRNAs) are duplicated in inverted repeat regions. The overall GC content of *P. longipedicellata* is 36.5% and in the LSC, SSC, and IR regions were 34.3%, 29.3%, and 42.4%, respectively.

Seven complete chloroplast genomes of Caryophyllaceae and two species from Amaranthaceae were used for constructing maximum likelihood method phylogenetic tree (Alignment was conducted using MAFFT v7.388 (Katoh and Standley 2013) and phylogenetic tree was constructed using IQ-TREE v1.6.6 (Nguyen et al. 2014)). The tree shows that genus *Pseudostellaria* is sister to Caryophylloideae, forming paraphyletic clade in Alsinoideae (Figure 1).

**Figure 1.**
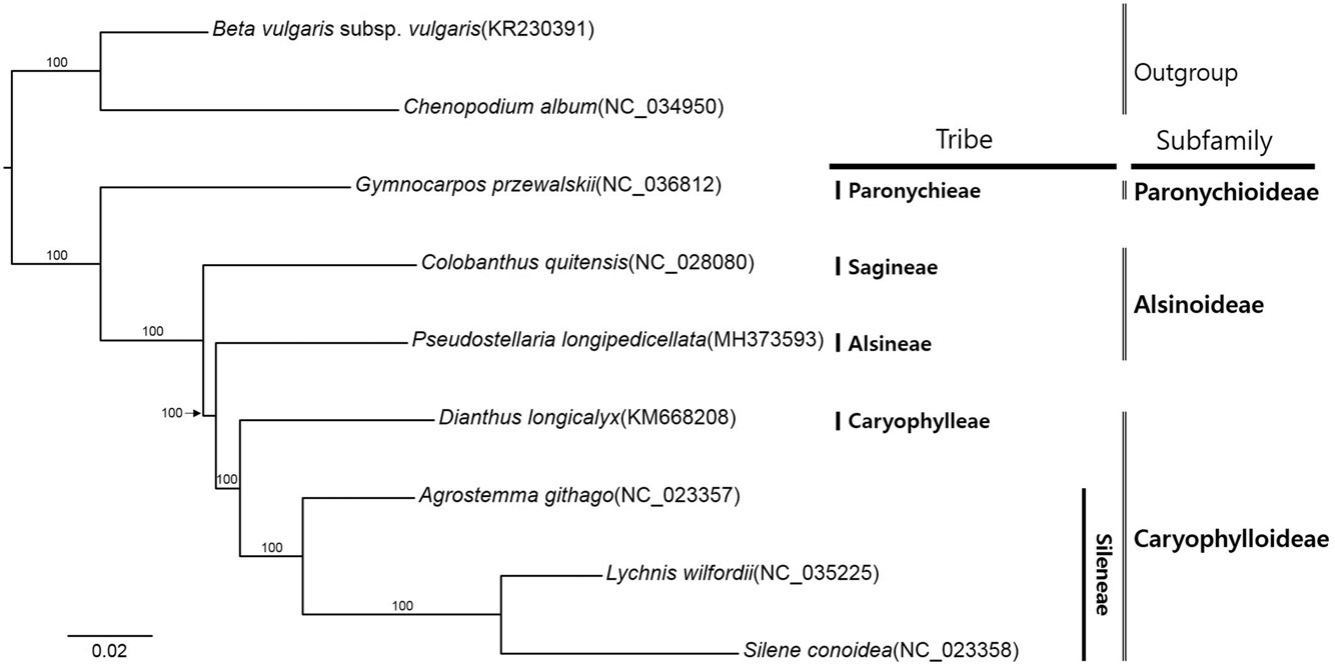
Maximum-likelihood phylogenetic tree of Caryophyllaceae based on nine complete chloroplast genomes. The numbers above branches indicate bootstrap support values.
